# Cancer and Smoking

**Published:** 1890-12

**Authors:** 


### CANCER AND SMOKING.

Since the death of President Grant, a constant smoker, cancer of the tongue and cigar smoking have been closely associated in the public mind. Surgeons of experience find that the disease is far more frequent in persons who have been in the habit of smoking. The disease appears to be about six times more common in males than in females. The affection known as "smoker's patch" is common. It is a slightly raised oval area on the forepart of the tongue, a little to one side of the middle line, just where the end of the pipe rests or where the stream of smoke from the pipe or cigar impinges on the surface of the tongue. The patch is usually red, but it may be bluish or pearly-white. It lasts for years, but tends to spread over the surface of the tongue if the irritation be continued. When diffused in this fashion, it constitutes leucoma of the tongue. Leucoma is certainly a predisposing cause of cancer. The smoker should never leave a "patch" untreated, and should avoid rough mouthpieces and brands of tobacco which cause irritation of the tongue.

